# *In vitro* Antibacterial Activity of *Ocimum suave* Essential Oils against Uropathogens Isolated from Patients in Selected Hospitals in Bushenyi District, Uganda

**DOI:** 10.9734/BMRJ/2015/17526

**Published:** 2015-05-08

**Authors:** Julius Tibyangye, Matilda Angela Okech, Josephat Maniga Nyabayo, Jessica Lukanga Nakavuma

**Affiliations:** 1 Department of Microbiology and Immunology, Faculty of Biomedical Sciences, Kampala International University, P.O. Box 71, Ishaka – Bushenyi, Uganda; 2 Department of Microbiology and Immunology, Faculty of Biomedical Sciences, Kampala International University, P.O. Box 71, Ishaka – Bushenyi, Uganda; 3 Department of Biomolecular Resources and Biolab Sciences, College of Veterinary Medicine, Animal Resources and Biosecurity, Makerere University, Kampala, P.O. Box 7062, Kampala Uganda

**Keywords:** Aromatic medicinal plants, bacteriauria, E. coli, resistance

## Abstract

**Aims:**

To determine antibacterial activity of *Ocimum suave* essential oils against bacterial uropathogens.

**Study Design:**

A cross sectional and experimental study.

**Place and Duration of Study:**

Six selected hospitals in Bushenyi District, Uganda between June 2012 and July 2013.

**Methodology:**

Clean catch midstream urine samples were collected and inoculated on Cystine Lysine Electrolyte Deficient (CLED) agar. The plates were incubated at 37°C for 24hrs to 48hrs. The *O. suave* essential oils were extracted by hydrodistillation of leaves for 4hrs using a Clevenger apparatus. The oil was collected and dried over anhydrous sodium sulphate (Na_2_SO_4_) and kept at 4°C till further use. The antimicrobial activity of *O. suave* essential oils against isolates was determined by agar well method. The MIC of *O. suave* essential oil extract was carried out by microbroth dilution method.

**Results:**

Of the three hundred (300) midstream urine samples collected, 67(22.33%) had significant bacterial growth. *Escherichia coli* is the most common isolate (61.19%, n = 41). The essential oil from *O. suave* showed activity against isolates of *E. coli, K. pneumoniae, S. aureus*, *E. feacalis, M. morganii, Citrobacter species, Enterobacter species* and *P. aeruginosa* with mean zone of inhibition (ZI) ranging from 10–22 mm. The essential oils had no inhibitory activity on *Acinetobacter species.* The minimum inhibitory concentration (MIC) for *O. suave* essential oils ranged from 0.78 to 22 μg/ml. This study showed that *O. suave* essential oils had MIC value of 0.78 μg/ml against *S. aureus* and MIC values ranging from 3 to 22 μg/ml against the other tested isolates.

**Conclusion:**

The most common uropathogen was *E. coli* (61.19% n = 41). *O. suave* essential oils exhibited antibacterial activity against majority of the uropathogens, except *Acinetobacter species,* mean ZI of 10–22 mm and MIC of 0.78 – 22 μg/ml.

## 1. INTRODUCTION

Urinary tract infections (UTIs) are commonly encountered conditions, especially in developing countries, with an estimated annual global incidence of at least 250 million [1–3]. About 150 million people worldwide are diagnosed with UTIs each year costing the global economy in excess of 6 billion US dollars [4–6]. The Uganda Bureau of Statistics, National Household Survey found the national prevalence of UTIs to be 0.2%. However, its impact and frequency vary in different populations [7]. The recent studies by Andabati and Byamugisha [8] found the prevalence at 13.3% and drug resistance occurrence of 20–62% in Mulago Hospital, Uganda.

*Escherichia coli* and other enterobacteriacae are the most common cause of UTIs and account for approximately 75% of the isolates [3]. The relative frequencies of the pathogens vary with age, sex, catheterization, and hospitalization [3,9]. Worldwide, *E. coli* causes 75–90% acute uncomplicated cystitis while *S. saprophyticus* accounts for 5–15%, mainly in younger women [4,10–12]. Although it is not always possible to trace the mode of entry of bacteria into the urinary tract, four possible routes of entry have been suggested; ascending infection; haematogenous spread; lymphogenous spread, and direct extension from another organ [13].

The search for antimicrobials of plant origin has been mainly stimulated by the fact that they contain multiple biochemical compounds to which microbes cannot develop resistance simultaneously. Due to occurrence of drug resistant uropathogens, there is a need to search for alternative and effective antimicrobial agents. Although, the drug resistance development by microbes cannot be stopped, appropriate use of more effective antibiotics including products of plant origin may reduce the mortality and health care costs [14,15]. Essential oils from aromatic medicinal plants have been reported by various researchers to exhibit antimicrobial activity against bacteria, yeasts, filamentous fungi, viruses and cancer [16–22]. However, there are few reports on its activity against uropathogens [22,23].

Traditional remedies utilizing plant products occupy the central place among rural communities in developing countries for curing various diseases, in the absence of an efficient primary health care system [15,24,25]. Traditional people, especially in the rural areas use *O. suave* for treatment of discomforts associated with the urinary and reproductive tracts. However, little information on the activity of this plant against uropathogens exists. Therefore, the aim of this study is to determine antibacterial activity of *O. suave* essential oils against uropathogens.

## 2. MATERIALS AND METHODS

### 2.1 Study Design

A cross sectional and experimental study was carried out in six selected health facilities (i.e. Kampala International University-Teaching Hospital (KIU-TH), Ishaka Adventist Hospital, Comboni Hospital, Bushenyi Medical Centre (BMC), Bushenyi Health Centre IV and Kyabugimbi Health Centre IV) in Bushenyi District, Uganda between June 2012 and July 2013.

#### 2.1.1 Inclusion and exclusion criteria

The study included patients aged 18 to 51 years attending out-and-in patient clinics, who were confirmed to have UTI signs and symptoms by the attending Clinician. All the patients with no history of antimicrobial drug administration for UTIs in the last two weeks and had consented to participate in the study.

The study excluded female patients who were in their menstruation period, patients aged below 18 years, those with history of antimicrobial drug administration in the last two weeks, non-Bushenyi residents, patients not registered at the selected hospitals and patients who had not consented to participate.

#### 2.1.2 Consent and counseling

A written consent was sought from the patients who satisfied the inclusion criteria. The Self-administered questionnaire and interview guide was carried out to capture demographic data, predicting factors for UTIs and counseling for specimen collection. The study subject was then sent for specimen collection and the results were kept confidential.

#### 2.1.3 Sample size and sampling procedure

Three hundred (300) morning midstream clean catch urine samples were collected from in-and-out patients with the help of trained nursing staff. The sample size (n) was calculated using the standard formula [26].

$$n=\frac{Z^{2}\text{QP}}{I^{2}}$$

Where: n = Sample size, Q = 100−P, Z = Level of significance (1.96) for confidence interval of 95%, P = Prevalence, I = margin of error of setting a significance level of 0.05 (i.e. 5%).

The urine samples were collected using random sampling method by taking the third patient on the waiting list of all the patients assessed for UTIs signs and symptoms by the attending Medical Officer or Clinician. Fifty samples were collected from each of the study areas with daily attendance of 150 patients with UTIs. The samples were then transported on ice to the laboratory for standard microbiological analysis within 30 minutes of collection. Baseline data such as patients’ age, sex, and clinical history were recorded at the time of sampling.

### 2.2 Isolation and Identification of the Uropathogens

Midstream clean catch urine samples were inoculated on CLED agar (Oxoid, UK) plates using a calibrated loop delivering 0.001ml of urine. Inoculated plates were incubated at 37°C for 24 to 48 hrs [27]. The samples were considered positive for UTI if pure culture of 10^5^CFU/ml were obtained from uncentrifuge urine sample and ≥5 pus cells observed in urine sample per field under microscope [8,28,29]. The presumptive identification of the isolates was based on the cultural characteristics on CLED agar (Oxoid, UK) plates and identification confirmed by standard identification protocol namely; Gram staining, motility test and conventional biochemical tests (i.e. oxidase, catalase, coagulase, IMViC, TSI agar, urease, gelatinase and the ability to grow in KCN [30–33]. The isolates were preserved using 15%v/v glycerol at −70°C.

### 2.3 Plant Collection and Identification

The leaves of *O. suave* were collected from Bushenyi District, South Western Uganda. Plant identification was carried out at the Department of Botany, Makerere University using plant shoots with leaves and flowers. Voucher specimen (JT 001) was deposited at the Makerere University Herbarium.

#### 2.3.1 Extraction of essential oils

Fresh mature leaves of *O. suave* were collected and thoroughly washed with distilled water twice. The excess water was drained off and the leaves cut into small pieces and hydro distilled for four (4) hours using a Clevenger apparatus. The oil was collected and dried over anhydrous sodium sulphate (Na_2_SO_4_). The extracted oil was stored at 4°C in glass bottle wrapped with aluminium foil.

A working solution of the essential oil was freshly prepared before use. Dimethyl sulfoxide (DMSO) was used to enhance oil solubility. A 100 μl of essential oil was diluted with 50 μl of dimethyl sulfoxide (DMSO) making a total volume of 150 μl. The working concentrations of essential oils were sterilized by filtering through a 0.2 μm single use filters (Sterile Acrodisc®).

### 2.4 Preparation of Bacterial Inoculums

The bacterial inoculum were prepared by suspending colony from a pure culture in sterile normal saline and the turbidity adjusted to match 0.5 McFarland standards; that is, about 5 × 10^5^CFU/ml. *Escherichia coli* ATCC 25922 and *S. aureus* ATCC 12692, obtained from Department of Medical Microbiology, Makerere University, were used as reference strains.

#### 2.4.1 Screening for antibacterial activity of essential oils

The antimicrobial activity of *O. suave* essential oils was screened against uropathogen isolates by the agar gel diffusion method [34], with slight modifications. Essential oil concentrations, ranging from 25 μg/ml to 50 μg/ml, were prepared with 0.5% DMSO. Ciprofloxacin and Nitrofurantoin were used as antimicrobial agent positive controls in the assay, while DMSO was the negative control.

#### 2.4.2 Minimum inhibitory concentration (MIC) of essential oils

The MIC reference of antimicrobial drug and *O. suave* essential oils extract was carried out by micro-broth dilution method using Mueller-Hinton broth (Oxoid, UK) [35–37]. Two-fold serial dilutions of essential oil, ranging from 5, 10, 20: 30, 40, and 50 μg/ml, were prepared with 0.5% DMSO. Then, for both the test and reference bacterial strains, 0.01ml of the standard isolate was inoculated into each well. The test was carried out in 96-well microtitre plates; 5 μl essential oil was dispensed into the first well containing 95μl of Mueller-Hinton broth and serially diluted by transferring 5 μl and 5 μl of inoculum added to each well. The plates were then incubated at 37°C for 18–24 h. The lowest concentration showing no visible growth was considered as the MIC.

### 2.5 Data Analysis

The data was entered in EpiData version 3.1.2701.2008, and statistical analysis was done by descriptive statistics using SPSS version 11.5. The antibacterial activity was reported in terms of diameters of the zones of inhibition (mm). The data was presented as mean ± standard deviation (SD). Comparison of means of zones of inhibition and MICs was done using student t-test and values of P < 0.05 were regarded as significant.

### 2.6 Ethical Approval

The ethical approval of the study was sought from Mbarara University of Science and Technology (MUST), Institutional Research and Ethics Committee (IREC) on Human Research, and Uganda National Council for Science and Technology (UNCST). All experiments were examined and approved by the appropriate ethics committees and performed in accordance with the ethical standards of the committees on human experimentation laid down in the Helsinki declaration of 1975 as revised in 2000.

## 3. RESULTS

**Fig 1:**
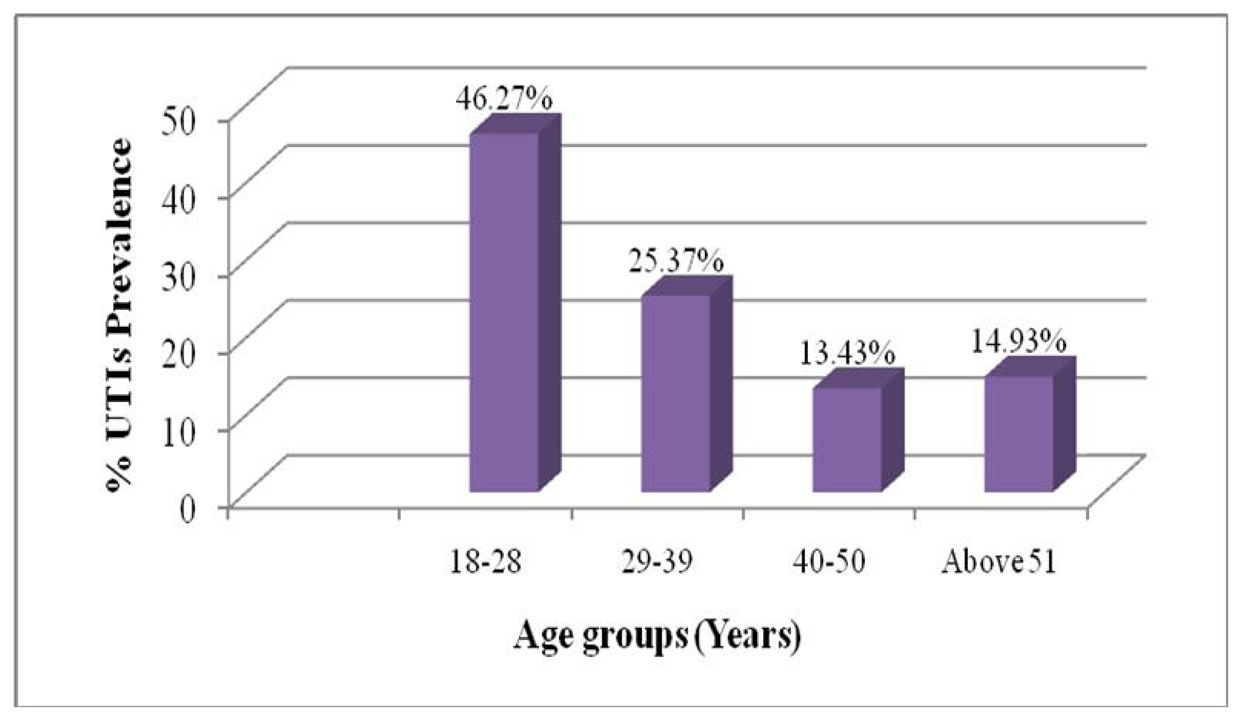
Prevalence of UTIs in different age groups

Three hundred (300) morning mid-stream clean catch urine samples were collected from patients attending the selected hospitals. Sixty seven samples 67 (22.33%) had significant bacteriuria. Of the 104 male urine samples, 22(21.15%) had positive cultures, compared to the 45(22.96%) out of 196 female samples. The prevalence of UTIs was found to be high (46.27%), in the age group of 18–28 years as presented in Fig. 1 below.

**Fig 2:**
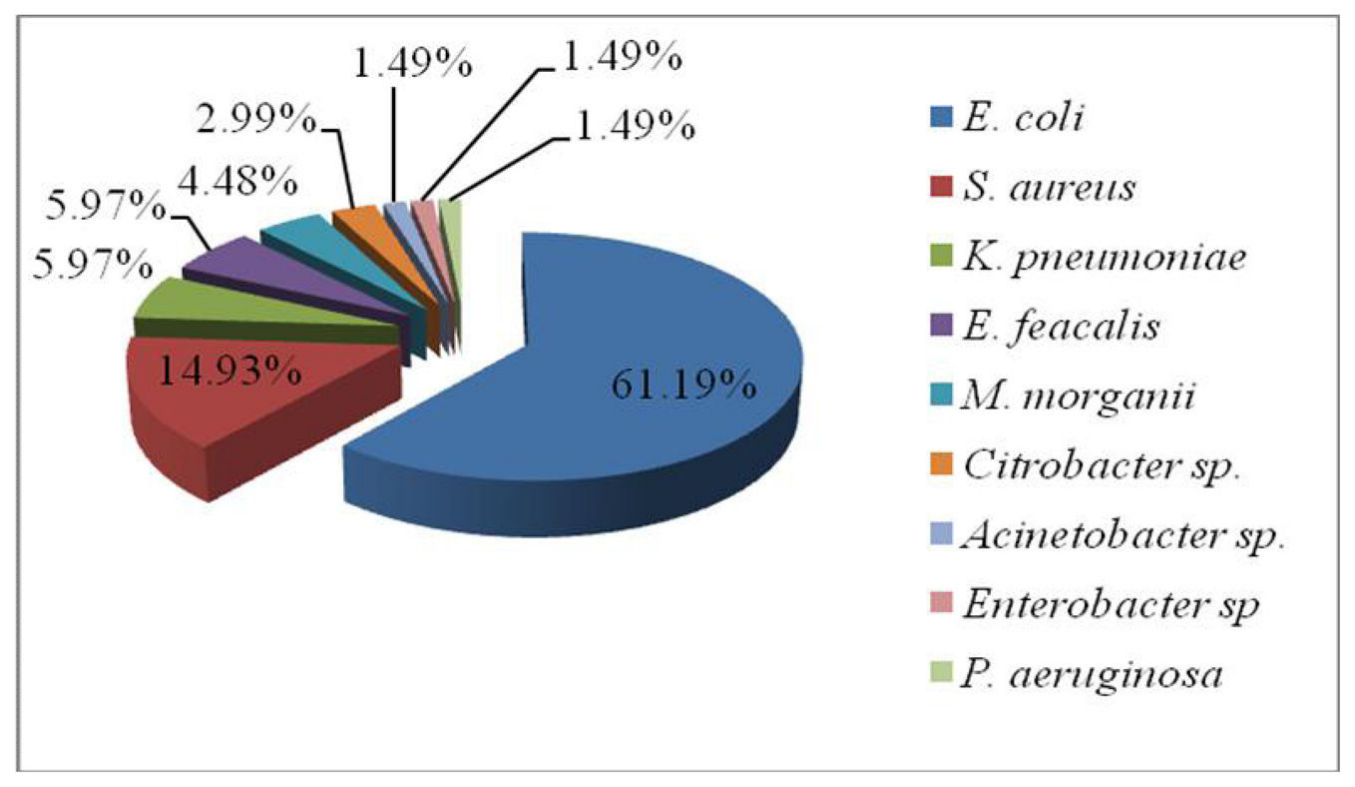
Percentage bacterial uropathogens isolated from patients

Urinary tract infections (UTIs) are mainly caused by bacteria and the findings in this study showed that nine bacterial uropathogens were isolated from 67 midstream clean catch urine samples of which *E. coli* was the most frequent isolate 41(61.19%), followed by *S. aureus*, 10(14.93%), *K. pneumoniae* 4(5.97%)*, E. feacalis* 4(5.97%)*, M. morganii* 3(4.89%), and *Citrobacter species* 2(2.99%). The least isolated were *Acinetobacter* species 1(1.49%)*, Enterobacter* species 1(1.49%), and *P. aeruginosa* 1(1.49%) as shown in Fig. 2 below.

Fresh leaves of *O. suave* yielded 0.2%v/wt of essential oil. The *O. suave* essential oil exhibited antibacterial activity against isolated uropathogens. The activity of essential oil was lower compared to the drug references used. The mean zones of inhibition ranged between 16–22 mm as compared to 13–29 mm and 11–26 for Ciprofloxacin (5 μg) and Nitrofurantoin (300 μg) reference antibiotic, respectively. The highest mean zone of inhibition (ZI) of 22 mm was exhibited against *E. coli*, whereas it was 23mm and 11 mm for ciprofloxacin and nitrofurantoin respectively against the same organisms. The activity of the essential oil was significant against the isolated of *E. coli,* mean ZI of 22 mm and 18 mm at 5% confidence interval, *P = .012*.

However, there was no significant difference in the activity of nitrofurantoin and essential oil with (*P = .786)*. The activity of essential oil showed no significant difference against the other uropathogens isolates when compared to ciprofloxacin (5 μg) and nitrofurantoin (300 μg) positive reference standard antibiotics. The mean ZI of essential oil were 23 mm ciprofloxacin and nitrofurantoin 15 mm against *S. aureus*. However, the essential oil showed no activity against *Acinetobacter species* and there was no inhibition of growth with the negative control (10% DMSO) as shown in Table 1 below.

The MICs for *O. suave* essential oils ranged from 0.78 to 22 μg/ml. This study revealed that *O. suave* essential oils showed maximum activity with MIC value of 0.78 μg/ml against *S. aureus* and showed moderate MIC values against the other test isolates. The minimum concentration (MBC) of antimicrobial necessary to kill an organism should be equal to or greater than the MIC for that microbe. In this study eight bacterial isolates presented MBCs which were within one two-fold dilution of the MIC obtained for the isolates. The MICs and MBCs of *O. suave* essential oils determined by the broth microdilution method of the isolated uropathogens are shown in Table 2 below.

## 4. DISCUSSION

Urinary tract infections (UTIs) are the most common infections that affects all age groups, men, women and children worldwide [13,38–40]. The results obtained were in line with the previous studies due to the fact that UTI signs and symptoms are not reliable indicators of the infection. Early diagnosis, timely and appropriate antimicrobial treatment are considered key factors for elimination of the uropathogens, prevent urosepsis and reduce the risk of renal scarring[13].

These findings are in agreement with most previous studies on UTIs [12,41]. UTIs due to *E. coli* are common in women because of its inherent virulence for urinary colonization particularly its adhesive abilities and the association with microorganisms ascending from the periurethral areas contaminated by fecal flora due to the close proximity to the anus and warm moist environment of the female genitalia[8]. The findings of this study are in line with the previous studies where similar results were observed by Tambekar et al. [42], reported significant bacteriuria in 39.1 of the cases, with the most common pathogens being *E. coli* (59%). The UTIs were found to be most frequent in female (63%) than male (37%) patients. According to Amin et al. [43], reported 68% females’ and 32% males’ urine cultures were positive for bacteria. The predominant isolate was *E. coli* with frequency rate of 59%. Other studies have also reported higher incidence of *E. coli* (47.30%) in urine samples [44]. It is interesting to note that only few have reported the presence of *Citrobacter* species, in UTIs [45,46].

The indiscriminate use of antimicrobial drugs has led to resistance in uropathogens globally [22]. Concurrent resistance to different antimicrobial agents has given rise to multi-drug resistance in uropathogens, which also complicates the therapeutic management of UTIs [4,22]. The spread of drug resistant uropathogens is one of the most serious threats to successful treatment of microbial diseases. Thus, the need for the discovery of new antimicrobial substances from natural sources, including plants. Essential oils from aromatic medicinal plants have been reported to exhibit antimicrobial effects against bacteria, yeasts, filamentous fungi, and viruses [21]. Essential oils are potential sources of novel antimicrobial compounds especially against bacterial pathogens [47]. *In vitro* studies in this study showed that *O. suave* essential oils inhibited bacterial growth. These findings are comparable to studies by Lima et al. [48], on *in vitro* antifungal activity of 13 essential oils obtained from plants against dermatophytes, *O. gratissimum* was found to be the most active, inhibiting 80% of the dermatophyte strains tested and producing zones of inhibition greater than 10mm in diameter. The MICs of the reference drugs used in this study were similar to those presented in [49].

The similar results were observed by Ilory et al. [50], on the antidiarrhoeal activities of leaf extracts of *O. gratissimum* with activity against *Aeromonas sobria*, *E. coli*, *P. shigelloides*, *S. typhi* and *S. dysenteriae*. The MIC for isolates ranged from 8 to 50mg/ml, while the MBC were from 8 to 62mg/ml. Other reports have shown MIC results similar to or higher [51,52]. These differences may be explained by susceptibility testing conditions, physicochemical characteristics of the oil, or even strain-to-strain differences and units of measurements.

According to Lopez et al. [53], *ocimum* oil has been described to be active against several species of bacteria and fungi. The chemical compositions in the essential oils are mainly of monoterpenes or sesquiterpenes with predominant features representing the terpenic chemotype group such as linalool and geraniol or the phenylpropenic chemotype groups, while the observed biological activities are attributable to either the individual components within the matrix of the oil or due to a synergistic effect of the components [54–60]. The prospect of further developing and using essential oils exhibiting broad spectrum biological activities holds promise in medicine and agriculture, owing to its low mammalian toxicity, biodegradability, non-persistence in the environment and affordability [60].

## 5. CONCLUSION

*E. coli* was the most common organism detected in this study. The *O. suave* essential oils showed activity against *E. coli, K. pneumoniae, S. aureus*, *E. feacalis, M. morganii, Citrobacter species, Enterobacter* species and *P. aeruginosa*; but had no activity against *Acinetobacter* species. The essential oils showed antibacterial activity against the isolated of *E. coli,* mean ZI of 22 mm and MIC of 0.78 μg/ml against *S. aureus*.

## Figures and Tables

**Table 1:** Antibacterial activity of *O. suave* essential oils (*p = < .05)*

Isolates	Mean inhibition zones (mm)				
	ID No.	CIP (5 μg/ml)	F (300 μg/ml)	EO (50 μg/ml)	DMSO
*E. coli*	KIUTH 010	22±0.58	18±0.58	16±0.00	0
*E. coli*	KHC 076	29±0.58	11±0.58	16±0.58	0
*E. coli*	KHC 053	13±0.58	18±0.58	21±0.58	0
*E. coli*	KHC 062	28±0.58	14±0.58	21±0.58	0
*E. coli*	IAH 148	23±1.00	11±0.58	22±0.58	0
*E. coli*	IAH 114	24±1.00	24±0.58	16±0.58	0
*E. coli*	IAH 112	25±0.58	21±0.58	16±0.58	0
*E. coli*	IAH 138	23±1.00	18±0.58	16±0.58	0
*E. coli*	CoH 122	26±1.00	26±0.58	17±0.58	0
*E. coli*	BMC 209	21±0.58	22±0.58	16±0.58	0
*E. coli* ATCC25922	**Control**	14±0.58	24±0.58	20±0.58	0
*S. aureus*	KIUTH 001	26±1.00	24±0.58	17±0.58	0
*S. aureus*	KIUTH 036	21±0.58	28±0.58	10±0.58	0
*S. aureus*	KIUTH 033	15±0.58	21±0.58	19±0.58	0
*S. aureus*	KIUTH 030	26±0.58	19±0.58	16±0.58	0
*S. aureus*	KIUTH 038	29±0.58	20±0.58	14±0.58	0
*S. aureus* ATCC12692	**Control**	14±0.58	18±0.58	10±0.58	0
*K. pneumoniae*	CoH 135	10±0.58	18±0.58	16±0.58	0
*K. pneumoniae*	BHC 093	22±0.58	13±0.58	14±0.58	0
*E. feacalis*	BHC 061	26±0.58	11±0.58	11±0.58	0
*M. morganii*	KHC 068	27±0.58	26±0.58	18±0.58	0
*Citrobacter species*	CoH 111	26±0.58	30±0.58	9±0.58	0
*Acinetobacter species*	IAH 129	24±0.58	20±0.58	-	0
*Enterobacter species*	KIUTH 026	17±0.58	16±0.58	16±0.58	0
*P. aeruginosa*	KHC 078	23±0.58	20±0.58	18±0.58	0

F – Nitrofurantoin, CIP – Ciprofloxacin, EO – Essential oil, DMSO - Dimethyl sulfoxide, - no activity

**Table 2:** Minimum bactericidal concentration (MBC) of *O. suave* essential oil

Isolate	ID No.	CIP	F	EOMIC (μg/ml)	EOMBC (μg/ml)
*E. coli*	KIUTH 010	1	31	6	12
*E. coli*	KHC 076	0.76	128	6	12
*E. coli*	KHC 053	4.5	32	13	13
*E. coli*	KHC 062	0.72	132	3	6
*E. coli*	IAH 148	0.95	30	3	6
*E. coli*	IAH 114	0.52	24	6	12
*E. coli*	IAH 112	0.82	24	6	12
*E. coli*	IAH 138	0.96	30	6	12
*E. coli*	CoH 122	0.91	29	7	7
*E. coli*	BMC 209	1	30	6	6
*E. coli* ATCC25922	**Control**	4.53	30	12	12
*S. aureus*	KIUTH 001	0.6	24	8	8
*S. aureus*	KIUTH 036	1	30	20	20
*S. aureus*	KIUTH 033	4.2	29	10	10
*S. aureus*	KIUTH 030	0.45	28	0.78	1.5
*S. aureus*	KIUTH 038	0.74	27	9	9
*S. aureus* ATCC12692	**Control**	4.25	26	11	11
*K. pneumoniae*	CoH 135	0.46	24	11	11
*K. pneumoniae*	BHC093	4.75	31	16	16
*E. feacalis*	BHC 061	0.46	24	9	9
*M. morganii*	KHC 068	0.9	29	8	8
*Citrobacter species*	CoH 111	0.8	27	6	12
*Enterobacter species*	KIUTH 026	0.87	27	11	11
*P. aeruginosa*	KHC 078	2	32	22	22

F – Nitrofurantoin, CIP – Ciprofloxacin, EO – Essential oil, EOMIC – Essential oil minimum inhibitory concentration, EOMBC – Essential oil minimum bactericidal concentration
